# Editorial: Design and bioapplication of nanozymes

**DOI:** 10.3389/fchem.2026.1785656

**Published:** 2026-01-29

**Authors:** Yue Cheng, Chuanhui Huang, Zhen Zhang

**Affiliations:** 1 State Key Laboratory of Advanced Materials for Intelligent Sensing, Key Laboratory of Organic Integrated Circuit, Ministry of Education and Tianjin Key Laboratory of Molecular Optoelectronic Sciences, Department of Chemistry, School of Science, Tianjin University, Tianjin, China; 2 Center for Advancing Electronics Dresden and Faculty of Chemistry and Food Chemistry, Technische Universität Dresden, Dresden, Germany

**Keywords:** antibacterial therapy, biomedical applications, biosensing and diagnostics, nanozymes, rational design

Nanozymes, nanomaterials with intrinsic enzyme-like catalytic activities, have rapidly evolved into a versatile class of functional materials with broad biomedical relevance (Zhang et al., 2024; Zhang et al., 2025). Advances in materials chemistry, nanotechnology, and biomedicine have enabled increasingly sophisticated nanozyme designs with enhanced catalytic performance and expanded application scopes (Tian et al., 2025). Rational nanozyme engineering and its translation into antibacterial therapy, biosensing, and disease diagnostics have attracted growing interest (Chen et al., 2023; Jiang et al., 2019). Accordingly, this Research Topic “*Design and Bioapplication of Nanozymes*” was established to highlight recent progress at the interface of nanozyme design and biological applications, with an emphasis on precision, intelligence, and clinical relevance.

The contributions collected in this Research Topic collectively reflect the current landscape of nanozyme research, emphasizing rational design, mechanistic understanding, and application-oriented innovation. Together, they illustrate the evolution of nanozymes from simple enzyme mimics into versatile biofunctional platforms.

A key theme highlighted in this Research Topic is the use of nanozymes as alternatives to conventional antibiotics to address the growing challenge of antimicrobial resistance (AMR). Wang et al. reported a ruthenium single-atom nanozyme (Ru-C_3_N_4_) with remarkable peroxidase-like activity for treating *Staphylococcus aureus*-induced otitis media. Atomic dispersion of Ru on a crystalline g-C_3_N_4_ support enabled near-maximal atomic utilization and enzyme-like catalytic efficiency comparable to natural horseradish peroxidase. This single-atom nanozyme exhibited narrow-spectrum antibacterial activity against Gram-positive bacteria, efficiently disrupted *S. aureus* biofilms, and alleviated inflammation *in vivo* while maintaining favorable biosafety. This work highlights how atomic-level engineering can simultaneously enhance catalytic activity, selectivity, and biocompatibility, providing a promising paradigm for nanozyme-based antibacterial therapies.

Complementing this experimental study, Zhao et al. provided a comprehensive review of antibacterial nanozymes, outlining their catalytic mechanisms, material categories, and synergistic therapeutic strategies. The review emphasizes that nanozyme-mediated antibacterial activity arises from multiple pathways, including reactive oxygen species (ROS) generation, membrane disruption, metal ion release, and photothermal effects. By surveying noble metals, metal oxides, carbon-based materials, MOFs, MXenes, and hybrid systems, the review underscores the structural diversity and tunability of antibacterial nanozymes. Moreover, the integration of nanozymes with photodynamic, photothermal, and sonodynamic therapies is discussed as an effective strategy to overcome the limitations of single-mechanism approaches. Together, these two contributions position antibacterial nanozymes as a mature yet rapidly advancing research frontier with strong translational potential.

Beyond antimicrobial applications, this Research Topic also highlights the growing role of nanozymes in biosensing and diagnostics, particularly through integration with molecular engineering and signal amplification strategies. Liao et al. developed an ultrasensitive amperometric immunoassay for detecting Epstein-Barr virus (EBV) latent membrane protein 1 (LMP-1) by combining AuPt alloy nanozymes with DNA tetrahedron nanostructures and strand displacement amplification. This design enabled efficient nanozyme capture and amplified electrochemical signalling, achieving high sensitivity, wide dynamic range, and reliable performance in biological samples. The study demonstrates how nanozymes can serve not only as catalytic labels but also as effective signal transducers, advancing nanozyme-based diagnostic platforms toward clinical relevance.

Intelligent and data-driven nanozyme design is emerging as a transformative direction, as highlighted by Park et al. in their review on machine learning (ML)-enhanced nanozymes. The authors show how ML can accelerate nanozyme development by predicting key structural and catalytic parameters, thereby reducing experimental trial-and-error. Integration of ML with colorimetric, electrochemical, and chemiluminescent nanozyme systems is enabling adaptive biosensing and real-time monitoring platforms. The review also discusses critical challenges, including data quality, model interpretability, scalability, and standardization, underscoring the need for continued methodological advances. Overall, the convergence of nanozymes and artificial intelligence opens new opportunities for smart and responsive bioanalytical and biomedical applications.

Taken together, the contributions in this Research Topic highlight several unifying themes: rational nanozyme design remains central to achieving high catalytic efficiency and functional specificity; nanozyme bioapplications are increasingly advancing from proof-of-concept studies toward disease-relevant and clinically meaningful scenarios; and interdisciplinary integration, particularly with DNA nanotechnology and machine learning, is reshaping nanozyme discovery and optimization.

Despite these advances, key challenges remain, including improving substrate specificity, ensuring long-term biosafety, elucidating *in vivo* catalytic behavior, and establishing standardized evaluation protocols. Addressing these issues will require continued interdisciplinary collaboration.

In conclusion, “*Design and Bioapplication of Nanozymes*” offers a timely overview of a rapidly evolving field. We hope this Research Topic informs readers of recent progress and inspires further innovation toward intelligent, precise, and translational nanozyme technologies. We thank all contributing authors and reviewers for their valuable efforts and anticipate that nanozymes will play an increasingly important role in future bioanalytical and biomedical applications.
